# The role of glymphatic dysfunction in cerebral small vessel disease and vascular cognitive impairment, and therapeutic implications

**DOI:** 10.1177/17474930261475465

**Published:** 2026-07-25

**Authors:** Hui Hong, Yutong Chen, Peiyu Huang, Hugh S Markus

**Affiliations:** 1 Stroke Research Group, Department of Clinical Neurosciences, University of Cambridge, Cambridge, UK; 2 Department of Radiology, The Second Affiliated Hospital of Zhejiang University School of Medicine, Hangzhou, China

**Keywords:** Cerebral small vessel disease, Glymphatic function, Treatment, MRI, Neuroimaging

## Abstract

**Background:**

The glymphatic system is a brain-wide perivascular clearance pathway that facilitates cerebrospinal fluid–interstitial fluid (CSF-ISF) exchange and the removal of metabolic waste from the brain parenchyma. Increasing evidence indicates that glymphatic transport is critically dependent on the integrity and function of cerebral small vessels, suggesting a close link with cerebral small vessel disease (cSVD). Structural and functional alterations of small vessels may disrupt glymphatic flow, while impaired glymphatic clearance may in turn exacerbate small vessel–related brain injury, indicating a bidirectional interaction between these two processes.

**Methods:**

In this review, we synthesize current evidence on the relationship between cSVD and glymphatic dysfunction, integrating findings from experimental models and in vivo human studies. We discuss shared pathophysiological mechanisms linking small vessel pathology to impaired perivascular fluid transport, summarize associations between glymphatic dysfunction and established cSVD imaging markers, and evaluate emerging therapeutic strategies aimed at restoring or enhancing glymphatic function. We highlight areas of convergence and inconsistency in the existing literature and identify key knowledge gaps that warrant further investigation. We also review potential therapeutic approaches, both behavioral and pharmacological to improve glymphatic function.

**Conclusion:**

Current evidence supports a close but incompletely understood bidirectional relationship between cSVD and glymphatic dysfunction. Targeting glymphatic function may represent a possible therapeutic strategy for cSVD and vascular cognitive impairment.

## Graphical abstract

**Graphical abstract:**
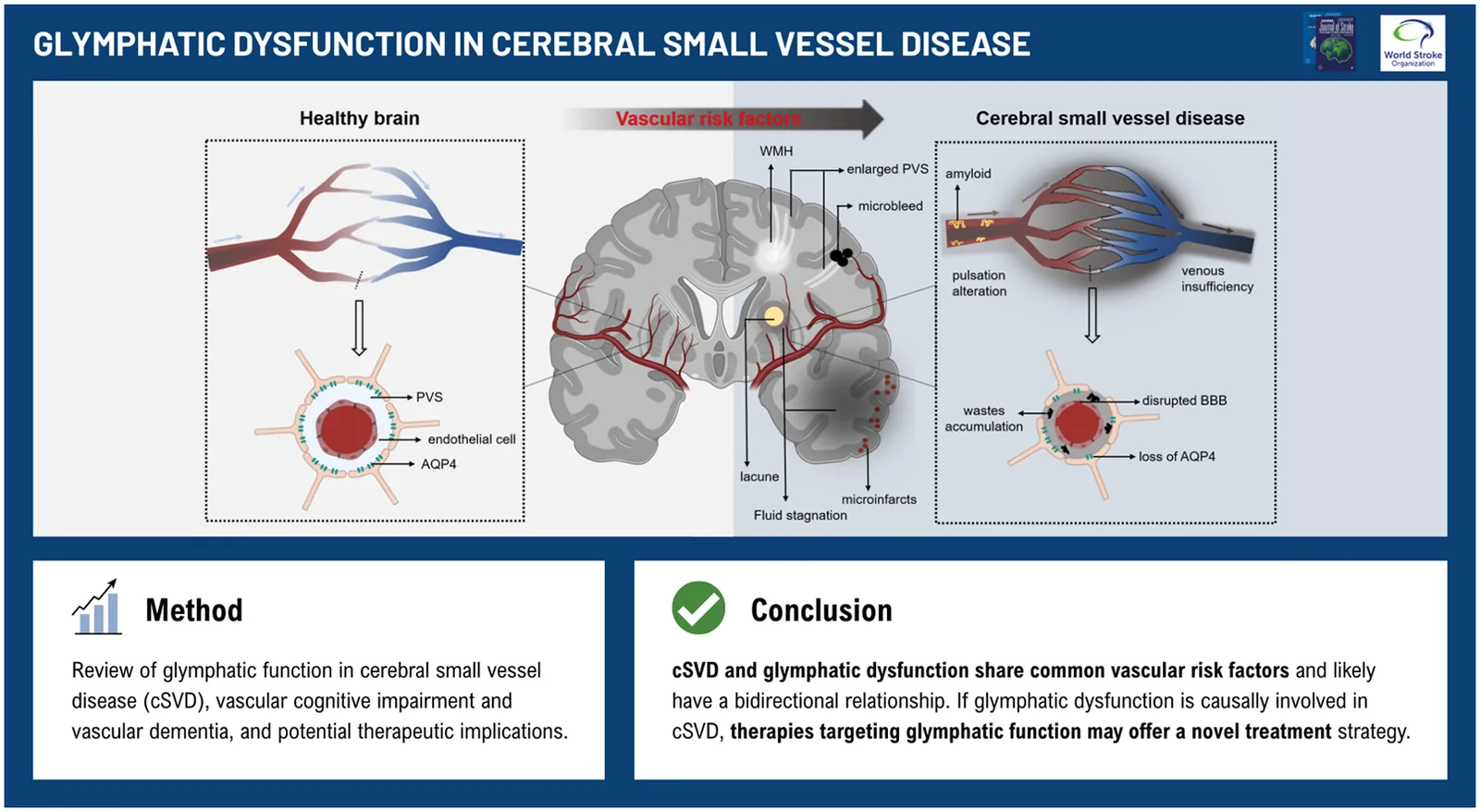

## Introduction

The glymphatic system is a recently described brain-wide perivascular clearance pathway that facilitates exchange of cerebrospinal fluid (CSF) and interstitial fluid (ISF), thereby removing metabolic waste products from the brain.^1^ Glymphatic transport occurs along perivascular spaces (PVS) which surround penetrating cerebral vessels. Flow is driven by arterial pulsation due to the cardiac cycle, respiration, and intrinsic slow vasomotion of the intracerebral vessels and mediated by aquaporin-4 (AQP4) water channels expressed on astrocytic endfeet.^2^ Experimental studies demonstrate that disruption of this system impairs the clearance of neurotoxic solutes, including amyloid-β and tau, highlighting its critical role in maintaining brain homeostasis and cognitive function^3^ (Figure 1).

**Figure 1.**
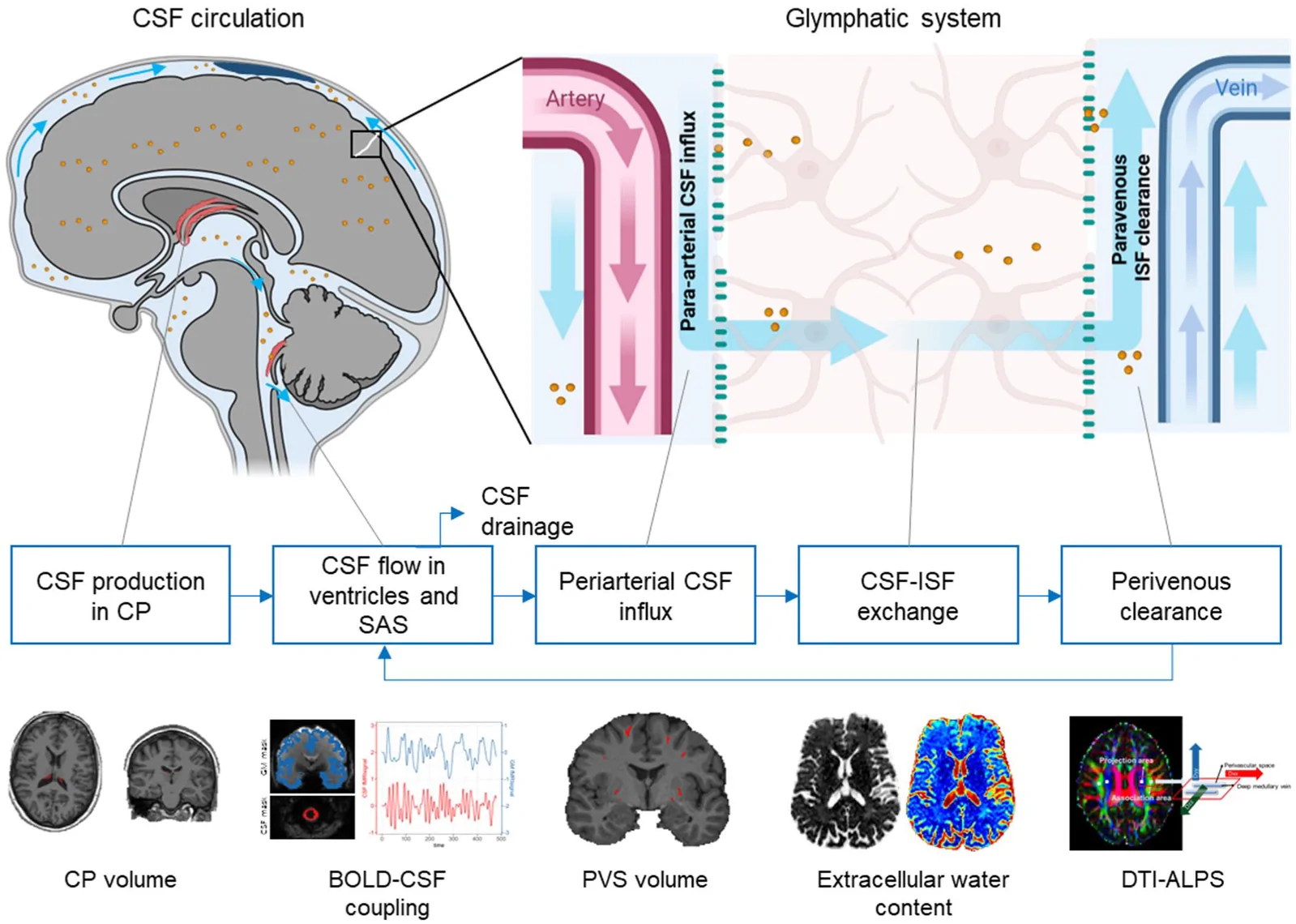
Schematic illustration of cerebrospinal fluid (CSF) circulation and glymphatic clearance pathways, with corresponding imaging markers for each process. CSF is primarily produced by the choroid plexus (CP), then circulates through the ventricular system and subarachnoid space (SAS). CSF subsequently enters the brain along the periarterial space, where it exchanges with interstitial fluid (ISF) within the parenchyma, facilitating solute transport and waste removal. The mixed CSF-ISF fluid is then cleared along the perivenous space and ultimately drained out of the brain. The lower panel shows representative imaging markers corresponding to different steps of this pathway, including CP volume, global blood oxygenation level–dependent (BOLD)-CSF signal coupling, PVS volume, extracellular water content, and diffusivity along the perivascular spaces (DTI-ALPS). Created in BioRender. Hong, H. (2026) https://BioRender.com/6trkwcf

Although some details of CSF drainage in humans remain controversial, a simplistic view is that CSF is produced primarily by the choroid plexuses (CP) and then enters the brain parenchyma from the subarachnoid space along the spaces surrounding arteries. CSF then moves into the brain tissue, mixing with ISF to wash away interstitial waste products. The CSF-ISF mixture moves toward the perivenous spaces to exit the brain tissue, and drains partially via the meningeal lymphatic vessels, and eventually reaches the cervical lymph nodes.

Cerebral small vessel disease (cSVD) represents a spectrum of pathological processes affecting small arteries, arterioles, capillaries, and venules of the brain.^4^ Conceptually, cSVD can be viewed as a continuum that begins with small vessel injury, and progresses to cSVD-related brain parenchymal damage, manifested as imaging markers such as white matter hyperintensities (WMH), lacunes, and cerebral microbleeds. Clinically these changes manifest as lacunar stroke, intracerebral hemorrhage, and vascular cognitive impairment and dementia.^5^

Given that glymphatic transport occurs predominantly along perivascular pathways, it is anatomically and physiologically intertwined with the cerebral small vessels.^6^ Therefore, structural and functional alterations of small vessels could directly disrupt glymphatic flow. Accumulating evidence from animal models and human imaging studies suggests that cSVD markers are associated with impaired glymphatic function. Conversely, glymphatic dysfunction may exacerbate small vessel–related brain injury by promoting ISF stagnation, impaired metabolic waste clearance, and increased vulnerability of the surrounding parenchyma. Together, these findings support a bidirectional interaction, in which small vessel pathology and glymphatic impairment may reinforce one another along the cSVD continuum.

This review provides a comprehensive synthesis of the emerging evidence linking cSVD and glymphatic dysfunction. Unlike previous reviews that described the glymphatic system in the broader context of neurodegenerative disorders, our review is cSVD-centric. We have systematically compiled the full spectrum of pathophysiological mechanisms linking cSVD and glymphatic impairment. In particular, we have dissected the potential causal directionality of each mechanism linking cSVD and glymphatic dysfunction. Furthermore, we integrate findings from both animal models and human studies, while assessing the strength and quality of the available evidence. By providing a structured synthesis and grading of current mechanistic and translational data, this review aims to clarify the current state of knowledge and identify key priorities for future research.

## Methodology

PubMed was searched using the terms (glymphatic OR perivascular space) AND (cerebral small vessel disease OR vascular cognitive impairment OR vascular dementia OR white matter hyperintensity OR lacune OR microbleed). Non-English and irrelevant studies were excluded, and only studies involving cSVD, vascular cognitive impairment, or vascular dementia were included.

## Glymphatic and CSF dynamics markers

In animal studies investigating glymphatic function (Table 1), the most commonly used approach is to assess the clearance of tracers injected into the brain, or into the CSF.^7^ Different imaging modalities are then employed to track tracer distribution and clearance. Real-time dynamics can be monitored using in vivo two-photon microscopy through a cranial window, allowing high spatial and temporal resolution and assessment of glymphatic transport, particularly in the cortical region.^8^ Alternatively, tracer distribution can be quantified postmortem using fluorescence microscopy on brain sections, enabling whole-brain mapping and integration with immunohistochemical markers such as AQP4.^9^ Contrast-enhanced magnetic resonance imaging (MRI), following intrathecal gadolinium administration, provides a complementary and less invasive approach, and allows dynamic and whole-brain assessment of glymphatic transport.^10^

**Table 1.** The summary of glymphatic and CSF dynamics markers.

Marker/technique	Evidence level	Specificity	Main biological process assessed	Major limitations
Intrathecal tracer monitor with two-photon microscopy	Animal tracer	Glymphatic transport	Direct CSF influx, CSF-ISF exchange, and clearance kinetics	Limited to animal models and cortical imaging
Postmortem fluorescent tracer mapping	Animal tracer	Glymphatic transport	Whole-brain tracer distribution and clearance	Terminal experiment; no longitudinal assessment
Contrast-enhanced MRI after intrathecal gadolinium (animal)	Animal tracer	Glymphatic transport	Dynamic whole-brain tracer movement	Requires invasive contrast administration
Contrast-enhanced MRI after intrathecal gadolinium (human)	Interventional human	Glymphatic transport	Direct visualization of CSF tracer movement	Invasive; limited clinical applicability
DTI-ALPS	Human proxy	CSF dynamics surrogate	Water diffusivity along presumed perivenous pathways	Influenced by white matter microstructure
Enlarged PVS burden/volume	Human proxy	CSF dynamics surrogate	Structural consequence of altered perivascular fluid movement	Strong dependence on MRI acquisition parameters, and WM-PVS may not reflect clearance
Global BOLD–CSF coupling	Human proxy	CSF dynamics surrogate	Coupling between neural/hemodynamic activity and CSF flow	Sensitive to physiological noise and acquisition protocol
Choroid plexus volume	Human proxy	CSF dynamics surrogate	Indirect marker of CSF production and clearance function	Mechanistic relationship remains uncertain
Extracellular/free-water imaging	Human proxy	CSF dynamics surrogate	Interstitial fluid accumulation	Poor specificity; affected by neurodegeneration and neuroinflammation
Meningeal lymphatic imaging	Human proxy	CSF dynamics surrogate	Lymphatic outflow pathways	Limited validation in cSVD
CSF-STREAM	Human proxy	CSF dynamics surrogate	PVS fluid mobility	Early-stage technique with limited clinical data

CSF: cerebrospinal fluid; ISF: interstitial fluid; DTI-ALPS: diffusivity along the perivascular spaces; PVS: perivascular spaces; BOLD: blood oxygenation level–dependent; CSF-STREAM: CSF-SelectiveT2-prepared REadout with Acceleration and Mobility-encoding.

In human studies, intrathecal administration of gadolinium-based contrast agents has also been used to visualize CSF dynamics.^11^ However, although this is often regarded as a gold-standard technique, its invasive nature limits its applicability, particularly in clinical settings and large-scale studies. To address this, several non-invasive MRI methods have been proposed to indirectly assess glymphatic alterations in vivo (Figure 1). These techniques reflect CSF dynamics rather than directly measuring glymphatic function. Here, we review the most widely used approaches.

The CP is the primary structure responsible for CSF production and also serves as an important interface for CSF-mediated waste clearance.^12^ Recent studies have suggested increased CP volume represents a marker of impaired glymphatic activity. In particular, increased CP volume has been linked to reduced CSF permeability and impaired waste clearance.^13,14^ Advances in neuroimaging analysis, particularly deep learning–based approaches integrating multiple MRI modalities, have enabled automated segmentation and quantification of the CP.^15^

Global blood oxygenation level–dependent (BOLD)–CSF signal coupling has been proposed as a metric reflecting CSF dynamics. This approach uses resting-state functional MRI (rs-fMRI) to simultaneously capture cerebral hemodynamic fluctuations associated with spontaneous neuronal activity and CSF inflow fluctuations from the spinal cord into the brainstem.^16^ Because CSF flow at the base of the brain is strongly influenced by physiological processes such as respiration and cardiac pulsation,^17^ sufficient temporal resolution is required to accurately characterize this coupling, which typically necessitates a short repetition time (TR) during rs-fMRI acquisition.

After reaching the brain surface, CSF enters via PVS, which appear hypointense on T1 and hyperintense on T2 MRI. Enlarged PVS likely reflect fluid accumulation and impaired CSF inflow, and are thought to predominantly represent periarterial rather than perivenous spaces.^18^ Earlier studies primarily relied on visual rating scales to assess PVS burden,^19^ whereas more recent work has used automated or AI-based segmentation methods to achieve more precise quantification.^20^ Reviews of the optimal PVS segmentation parameters were available elsewhere.^21,22^ Briefly, more accurate PVS quantification is achieved with higher magnetic field strength (7T) and three-dimensional (3D) acquisition, which achieves higher spatial resolution and reduces partial volume effects.^21^ Most segmentation methods were developed on T2-weighted, or T1-weighted MRI, or combination of the two.^21,22^ Several studies reported good generalizability across centers.^23,24^

Within the brain parenchyma, delayed CSF-ISF exchange may lead to macromolecule accumulation, altered osmotic pressure, and increased ISF volume, detectable by white matter free water using single shell diffusion MRI,^25^ or isotropic volume fraction using the Neurite Orientation Dispersion and Density Imaging (NODDI) method.^26^ This can also be quantified using diffusion spectra analysis to isolate an ISF compartment in intravoxel incoherent motion MRI.^27^ However, extracellular water is influenced by vascular and neurodegenerative processes, including cSVD-related white matter damage, limiting its specificity.^25^

Diffusivity along the perivascular spaces (DTI-ALPS) is increasingly used as a non-invasive technique of assessing glymphatic function. It uses diffusion MRI to evaluate movement of water molecules along PVS, which are presumed to run parallel to the deep medullary veins (DMVs). Early studies manually placed regions of interest areas corresponding to the DMVs,^28^ but more recent studies have adopted automated quantification approaches, enabling standardized assessment and facilitating analyses in large cohorts.^29,30^ Numerous studies have reported cross-sectional associations between DTI-ALPS and clinical and cognitive function across various neurological disorders.^31–33^ Furthermore, a few prospective studies demonstrate it predicts cognitive decline and dementia, independent of conventional DTI markers such as mean diffusivity (MD).^29,33,34^

Despite the advent of a variety of non-invasive markers of CSF dynamics, to date, we are not aware of any studies directly validating CP volume, BOLD-CSF coupling, or free water against clearance of intrathecally administered gadolinium. The DTI-ALPS index was found to correlate with measurements made using intrathecal gadolinium contrast in humans, validating the technique,^35^ although a subsequent similar study has cast doubt on whether DTI-ALPS reflects clearance of gadolinium contrast in the perivenous region.^36^ Furthermore, it was possible that DTI-ALPS was confounded by altered white matter structural integrity.^37^ As for PVS volume, white matter PVS exhibited delayed and variable enhancements after intrathecal administration of gadolinium, raising concerns that white matter PVS may not reflect glymphatic clearance.^38^ As for free water, its measurement could be confounded by neuroinflammation,^39^ rather than reflecting extracellular fluid stagnation and reduced clearance.

A number of more recent techniques to image CSF dynamic have been proposed, including imaging of meningeal lymphatics,^40^ and a CSF-specific MRI technique, CSF-SelectiveT2-prepared REadout with Acceleration and Mobility-encoding (CSF-STREAM), which allows fluid mobility in the PVS to be assessed in humans,^41^ but as yet has been limited application of these to cSVD.

## Vascular risk factors and their associations with glymphatic dysfunction

Vascular risk factors, particularly hypertension, are key risk factors for cSVD, and many studies have reported associations between these risk factors and markers of impaired CSF dynamics.

### Hypertension

Chronic elevation of blood pressure, particularly systolic pressure, has been associated with enlarged PVS in the basal ganglia,^42,43^ reduced DTI-ALPS, reduced BOLD-CSF coupling, and increased CP volume.^29,44^ Animal models demonstrate hypertension impairs arterial pulsatility and perivascular compliance, thereby slowing glymphatic CSF inflow and solute clearance.^45^ Together, these findings support a mechanistic model in which hypertension-induced vascular stiffening impairs perivascular drainage pathways of the glymphatic system, leading to PVS enlargement.^46^

### Diabetes

In human studies, type 2 diabetes has been associated with lower DTI-ALPS, lower BOLD-CSF coupling,^44^ and higher CP volume.^29^ In longitudinal studies, it was associated with a faster decline in BOLD-CSF coupling.^44^ Both diabetes and insulin resistance in non-diabetic individuals are associated with enlarged PVS in the basal ganglia and centrum semiovale.^44,47^

### Hyperlipidemia

Lipid metabolic dysfunction has been associated with glymphatic disturbance and cSVD. Visceral fat and higher triglyceride glucose index TyG (a triglyceride–glucose proxy of insulin resistance) are associated with greater centrum semiovale enlarged PVS,^48,49^ and peripheral apolipoproteins (apoB, apoB/apoA-1, apoE) independently track PVS severity.^50^ Using Mendelian randomization, lipid-lowering via NPC1L1 (the target of ezetimibe) was associated with reduced PVS burden and small vessel stroke risk, implying a potentially causal, targetable lipid pathway.^51^

### Other vascular risk factors

Among healthy aging populations, smoking was associated with increased PVS volume,^52^ decreased DTI-ALPS, decreased BOLD-CSF coupling, and increased CP volume.^29^ Serum homocysteine level is associated with increased PVS volume in cSVD,^53^ and PVS volume mediates the relationship between homocysteine and cognitive impairment.^54^

Therefore, vascular risk factors, which are associated with cSVD, also appear closely linked to glymphatic impairment, likely via heterogeneous mechanisms. For example, hypertension may affect small vessel mechanics and pulsatility, whereas diabetes may impair glymphatic function through endothelial dysfunction and blood–brain barrier (BBB) disruption.

## The bidirectional relationship between cSVD and glymphatic function

Structural and functional alterations in small cerebral vessels—including changes in pulsatility, cerebrovascular reactivity (CVR), BBB permeability, venous insufficiency, and vascular amyloid deposition—may impair glymphatic function. Conversely, glymphatic dysfunction may promote cSVD progression, including WMH development and vascular amyloid accumulation, indicating a bidirectional relationship (Figure 2).

**Figure 2.**
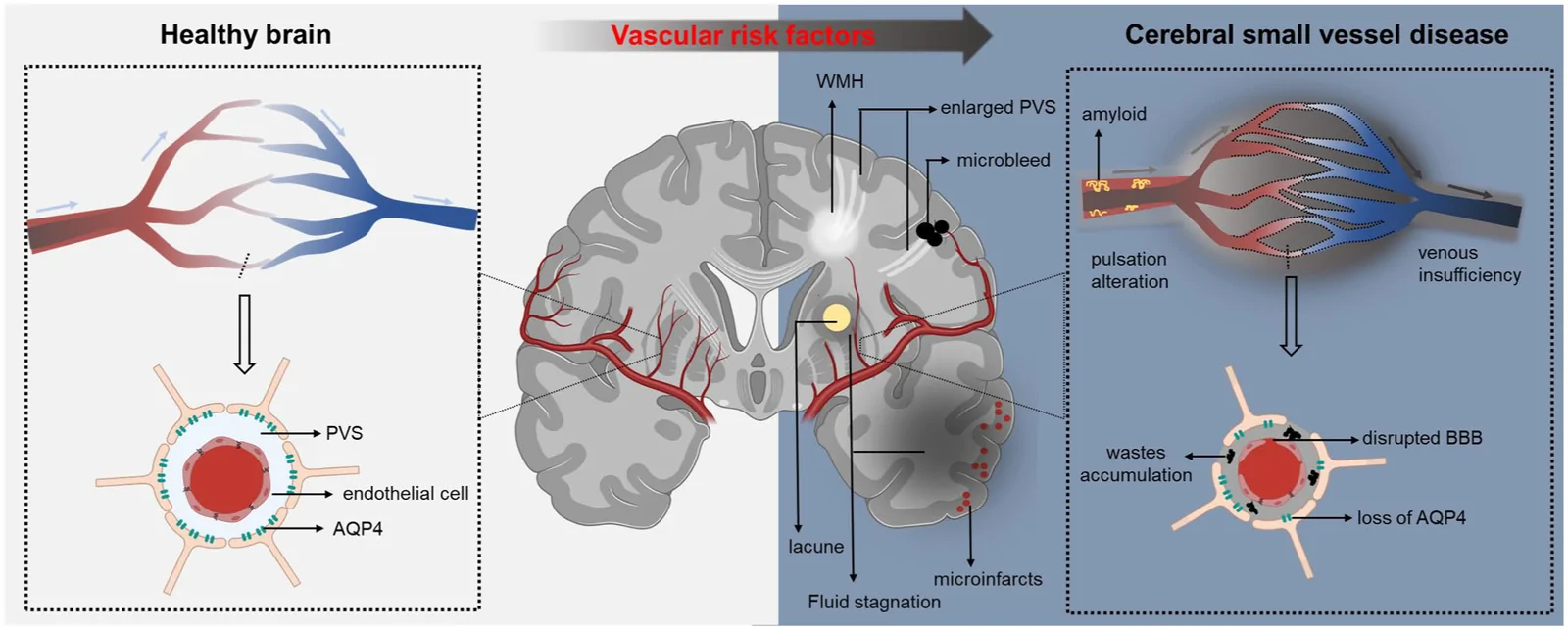
Bidirectional relationship between cSVD and glymphatic function. In the healthy brain, intact vascular structure supports efficient fluid transport along perivascular spaces (PVS), facilitating movement from the arterial to the venous system (left panel). With vascular risk factors, structural and functional alterations in small cerebral vessels occur, including changes in vascular pulsatility, impaired cerebrovascular reactivity (CVR), amyloid accumulation, blood–brain barrier disruption, and venous insufficiency. These changes contribute to impaired glymphatic function, manifested by PVS enlargement and interstitial fluid stagnation within the brain parenchyma. In addition, cSVD-related brain injuries—such as white matter hyperintensities (WMH), cerebral microbleeds, lacunes, and microinfarcts—may further exacerbate glymphatic dysfunction. Conversely, glymphatic impairment, particularly enlarged PVS, may also contribute to the development and progression of WMH (right panel). Created in BioRender. Hong, H. (2026) https://BioRender.com/32gc46n

### Vascular pathophysiological processes resulting in glymphatic impairment

Pulsatility represents an important vascular mechanical property and is a key physical driver of the brain’s glymphatic system. Cardiac-driven arterial pulsation causes PVS to cyclically expand and contract, thereby facilitating the inward movement of CSF and the outward clearance of ISF. Evidence from animal studies demonstrates that decreased arterial pulsatility is associated with impaired CSF flow along PVS and weakens CSF-ISF exchange,^55^ although data from human studies are less consistent. Nevertheless, a recent longitudinal study suggests that higher baseline pulsatility was associated with reduced PVS enlargement.^56^

CVR is primarily determined by the ability of the cerebral small vessels to dilate and can be measured using either functional MRI or arterial spin labeling to measure reactivity of cerebral blood flow to a hypercapnic challenge. Impaired CVR has been associated with enlarged PVS, slower CSF dynamics, and higher cSVD burden.^57,58^ In both cross-sectional and longitudinal analyses, lower baseline CVR predicted progression of WMH and PVS over time, reinforcing its role as an early driver of structural damage.^58,59^

The BBB and glymphatic system were thought to form a complementary mechanism in the transport and clearance of interstitial solutes and fluid.^60^ While the BBB mediates selective exchange between the blood and brain parenchyma, the glymphatic system facilitates the removal of excess protein waste, metabolites, and solutes that are less efficiently cleared via BBB-dependent mechanisms. However, in cSVD, evidence linking BBB and glymphatic dysfunction has been mixed with some studies showing increased BBB permeability was associated with enlarged PVS, and others showing no association.^61,62^

Emerging research suggests that impaired venous drainage, especially involving DMVs, plays a role in the disruption of glymphatic circulation in cSVD. The venous system not only clears deoxygenated blood but also provides critical conduits for interstitial and CSF outflow, functioning in parallel with the glymphatic network.^63^ Reduced DMV count, visibility, or continuity on susceptibility-weighted imaging correlates with markers of compromised glymphatic functions (enlarged PVS and/or decreased DTI-ALPS) as well as greater cSVD burden.^64,65^

### Impact of glymphatic impairment on CSVD lesion formation

Animal models with multiple microinfarcts have shown that solutes derived from CSF become trapped around ischemic lesions, accompanied by reduced AQP4 expression.^9,66^ Human studies using various imaging markers have demonstrated associations between glymphatic impairment and the presence and number of lacunes.^34,35^ Regional analyses revealed increased water accumulation surrounding lacunes, indirectly supporting lesion-related disruption of local glymphatic function.^67,68^

Traditionally, WMH has been explained by chronic hypoperfusion.^69^ However, increasing evidence supports a fluid stagnation hypothesis, in which WMH reflects ISF stagnation.^70^ Several studies have demonstrated a close spatial relationship between PVS and WMH.^71,72^ These findings suggest that glymphatic dysfunction, manifested as PVS enlargement, may contribute to the development and progression of WMH.

### Interaction between amyloid dysfunction and glymphatic impairment

Increasing research highlights the interplay between amyloid deposition and glymphatic dysfunction in cSVD, particularly the cerebral amyloid angiopathy (CAA) subtype. Enlarged PVS have been associated with amyloid-related biomarker abnormalities, including amyloid-positive CSF Aβ_1-42_ status and a lower plasma Aβ42/40 ratio, suggesting a potential link between PVS alteration and amyloid pathology.^73,74^ Consistently, higher PVS volumes have been observed in carriers of CAA-related mutations,^75^ and greater cortical amyloid deposition in patients with CAA has been linked to enlarged PVS in the centrum semiovale.^76^ Pathologically, amyloid deposition in cortical vessels causes smooth muscle loss, reducing pulsatility and perivascular clearance, leading to PVS dilation.^77^

In line with this mechanism, lobar cerebral microbleeds, a characteristic imaging marker of the CAA subtype of cSVD, have been associated with PVS enlargement in the centrum semiovale.^78^ A higher burden of lobar microbleeds correlates with increased PVS enlargement,^79^ and spatial analyses demonstrated colocalization between enlarged PVS and adjacent microbleeds.^80^ Together, these findings suggest a bidirectional cycle where amyloid pathology impairs glymphatic clearance and enlarges PVS, while reduced clearance promotes further amyloid deposition.

## The role of inflammation and sleep in connecting cSVD and glymphatic function

### Inflammation

Growing evidence indicates that both systemic and central nervous system (CNS) inflammation play a key role in disrupting the glymphatic circulation in cSVD. PVS enlargement is associated with upregulation of adhesion molecules such as vascular cell adhesion molecule 1 (VCAM-1), P-selectin, and intercellular adhesion molecule 1 (ICAM-1),^81,82^ while E-selectin appears protective against PVS enlargement.^83^ These findings suggest vascular inflammation may impair perivascular drainage through endothelial activation and remodeling of the vessel wall. PVS enlargement is also correlated with systemic inflammatory indices such as the neutrophil-to-lymphocyte ratio,^84^ C reactive protein,^85^ and pro-inflammatory cytokines such as tumor necrosis factor (TNF)-related apoptosis-inducing ligand (TRAIL) and interleukin (IL)-18.^83^ Anti-inflammatory cytokines such as IL-1RL2, IL-3Ra, and IL-5Ra appear to be protective.^83^ Similarly, in CADASIL, inflammatory proteins related to leukocyte migration (e.g. IL-8, MCP-1) are associated with both PVS volume and white matter injury, suggesting shared inflammatory–glymphatic mechanisms across genetic and sporadic forms.^86^ In terms of inflammation within brain parenchyma, high-resolution imaging shows increased microglial activation around enlarged PVS, suggesting local neuroinflammation contributes to glymphatic stasis.^61^

### Sleep

In animal studies, glymphatic clearance has been shown to be increased during sleep,^87,88^ while small studies with intrathecal gadolinium injection support a similar relationship in humans.^89^

In contrast, the relationship between sleep and cSVD remains more controversial. Studies examining sleep disorders such as obstructive sleep apnea and insomnia have reported inconsistent findings, with both positive and negative associations observed with imaging markers of cSVD.^90–92^ While some studies have suggested that longer sleep duration is associated with an increased risk of cSVD, others have conversely reported that shorter sleep duration is linked to higher cSVD burden.^93,94^

Recent mechanistic studies suggest that cSVD itself may attenuate sleep-related glymphatic activity. Increasing evidence highlights the role of vasomotion, particularly slow oscillations around 0.1 Hz, in driving solute clearance during non-rapid eye movement (NREM) sleep.^88,95^ The micro-architectural organization of NREM sleep, shaped by norepinephrine fluctuations and vascular dynamics, was reported to be a key determinant of glymphatic clearance.^88^ Notably, cSVD has been associated with reduced vasomotion amplitude,^96^ suggesting a potential mechanism by which vascular dysfunction may impair sleep-dependent glymphatic clearance.

## The association between glymphatic function and cSVD-related vascular dementia

Studies using DTI-ALPS have consistently reported associations with vascular cognitive impairment and suggest that the DTI-ALPS index may predict the risk of vascular dementia.^33,34^ CP volume and BOLD-CSF coupling have also been associated with cognitive impairment.^29^

Most studies are cross-sectional, raising concerns that glymphatic markers may simply reflect more severe cSVD. However, recent studies controlling for other MRI markers and using prospective follow-up have addressed this. In the SCANS cohort of patients with lacunar stroke and confluent WMH, higher baseline DTI-ALPS predicted a lower risk of dementia, remaining significant after adjustment for median MD.^33^ Change in DTI-ALPS over 3 years predicted dementia conversion over 5 years, but when median MD was entered as a covariate, the association was not significant. The conclusion was that DTI-ALPS may predict future dementia risk, but that part of the association may be due to the DTI-ALPS signal also representing microstructural damage. In about 40,000 community individuals in UK Biobank, lower DTI-ALPS, lower BOLD-CSF coupling, and higher CP volume all predicted incident dementia over a median 5.3 years of follow-up.^29^ These results remained significant after adjusting for white matter MD, cardiovascular risk factors, and APOEε4 carrier status. Both DTI-ALPS and CP volume mediated the effect of WMH and diabetes duration on dementia.

In contrast, most studies, including a meta-analysis of five population-based cohorts, have found no association between enlarged PVS and cognitive decline, or future dementia risk.^33,97^ Similarly, in the above-mentioned UK Biobank study, PVS was the only one of the four CSF dynamic markers not to predict future dementia risk. These differences are likely to reflect the fact that these imaging markers reflect different components of CSF dynamics (see Figure 1).

## Impaired glymphatic function—a possible link between cSVD and neurodegeneration diseases

cSVD rarely occurs in isolation in the aging brain and frequently coexists with neurodegenerative pathologies.^98^ The presence of cSVD pathology has been shown to greatly increase the probability that AD pathology will result in clinical dementia during life.^99^ Therefore, beyond its role in vascular cognitive impairment, impaired glymphatic function may represent a potential mechanistic link between cSVD and neurodegenerative diseases.

If cSVD disrupts glymphatic transport, this would be expected to impair clearance of neurotoxic proteins, including amyloid-β and tau. This provides a plausible biological link between cSVD and neurodegenerative disorders. In AD patients with CAA, a subtype of cSVD, the perivascular drainage of amyloid-β peptides from the brain was impaired.^100^ More recent in vivo imaging studies further support this hypothesis, showing that reduced DTI-ALPS indices mediate the association between cSVD burden and the accumulation of amyloid and tau pathology.^32,101^

Beyond AD, similar interactions between cSVD and impaired glymphatic clearance have been proposed in other neurodegenerative diseases. For example, α-synuclein–related disorders such as Lewy body disease,^102^ as well as astrocytic activation in frontotemporal dementia,^103^ may also involve glymphatic dysfunction that contributes to abnormal protein accumulation and disease progression.

Taken together, these findings suggest cSVD may contribute to the development and progression of multiple neurodegenerative diseases, and therefore targeting vascular health and glymphatic function could represent a strategy to mitigate both vascular and neurodegenerative brain pathologies. More research and clinical trials in this area are required.

## Therapeutic implications

A role for glymphatic impairment in cSVD raises the exciting possibility that therapeutic strategies that improve glymphatic function could represent treatments for cSVD (Table 2).

**Table 2.** Candidate glymphatic-directed therapies for cSVD.

Shared mechanisms of glymphatic function and cSVD	Intervention	Main mechanism	Evidence level	Most relevant cSVD subtype	Key evidence
Vascular risk factors	Intensive blood pressure control	Reduces vascular injury	Randomized controlled trials (RCT)	Hypertensive arteriopathy	Reduced perivascular space (PVS) volume fraction in randomized trial
	Calcium channel blockers (CCBs)	Reduces vascular injury	RCT	Hypertensive arteriopathy	Lower PVS burden associated with CCBs exposure
Vascular amyloid accumulation	Mesencephalic astrocyte-derived neurotrophic factor (MANF)	Promotes blood vessel Aβ removal	Animal study	Potentially cerebral amyloid angiopathy (CAA)	Improved glymphatic function and reduced Aβ deposition in the brain blood vessels after ischemic injury
Sleep	Melatonin	Improves circadian regulation and aquaporin-4 (AQP4) polarization	Animal study	Not mentioned	Increased glymphatic activity and AQP4 polarization
	Dexmedetomidine	Reduces noradrenergic tone; restores AQP4 polarity	Animal study	Not mentioned	Increased CSF-ISF exchange and tracer transport
	Adrenergic antagonist cocktail	Suppresses adrenergic signaling	Animal study	Not mentioned	Enhanced vasomotion, followed with increased glymphatic flow
	Orexin receptor antagonists	Regulator of wakefulness	Animal study/in-human study	Not mentioned	Increased protein clearance; high vasomotor activity in human model of orexin deficiency
Inflammation	β-hydroxybutyrate	Suppresses microglial activation and neuroinflammation	Animal study	Not mentioned	Enhanced glymphatic drainage in subarachnoid hemorrhage (SAH) model
Potentially others (neuromodulation)	Continuous theta burst stimulation (cTBS)	Potentially neuromodulation of glial activity	RCT	Not mentioned	Improved glymphatic measures and cognition in cSVD patients
	Focused ultrasound with microbubbles	Amplify vasomotion	Animal study	Potentially CAA	Improved clearance of Aβ; improved memory

Controlling vascular risk factors may improve glymphatic function, although direct interventional evidence remains limited. A randomized trial comparing intensive systolic blood pressure control (<120 mm Hg) with standard treatment (<140 mm Hg) found that intensive therapy reduced PVS volume fraction.^104^ The study also found that lower PVS volume fraction was associated with exposure to calcium channel blockers (CCBs), while a Mendelian randomization study showed that genetically proxied CCB use was associated with reduced PVS volume in the basal ganglia.^105^ Consistent with this, other Mendelian randomization studies suggested CCBs may be more effective in treating cSVD than other antihypertensive agents.^106^

Reducing vascular Aβ accumulation may represent a potential strategy for improving glymphatic function in the CAA subtype of cSVD. Parenchymal border macrophages (PBMs) play an important role in regulating glymphatic function. In an animal model of ischemic stroke, supplementation with mesencephalic astrocyte-derived neurotrophic factor preserved PBMs, enhanced glymphatic function, and reduced vascular Aβ deposition following ischemic injury.^107^

Improving sleep may enhance glymphatic function in cSVD. Melatonin, an endogenous circadian regulator, may promote glymphatic activity by modulating circadian proteins and enhancing AQP4 polarization in astrocytic endfeet.^108,109^ In contrast, zolpidem, a non-benzodiazepine hypnotic, induces sedation but has been reported to suppress glymphatic activity, potentially by reducing infraslow EEG sigma oscillations and associated norepinephrine dynamics during NREM sleep.^88^ Pharmacological modulation of the noradrenergic system represents another potential strategy. Reduced noradrenergic tone during sleep is a key factor underlying increased glymphatic clearance.^87^ In animal studies, dexmedetomidine, a selective α2-adrenergic agonist, restored polarized AQP4 localization in astrocytic endfeet and facilitated CSF-ISF exchange and glymphatic transport.^110^ Similarly, acute administration of an adrenergic antagonist cocktail (propranolol, prazosin, and atipamezole) enhanced glymphatic flow.^111^ However, clinical translation remains uncertain, and many noradrenergic agents carry cardiovascular side effects. In addition, orexin receptor antagonists, which promote sleep by blocking the orexin system, a key regulator of wakefulness, have been shown to reduce ISF Aβ levels, potentially through enhanced glymphatic clearance.^112,113^ Recent studies in human models of orexin deficiency have also demonstrated increased vasomotor activity, further supporting a potential role for orexin signaling in glymphatic regulation through modulation of vasomotion.^114^ Nevertheless, direct evidence linking orexin inhibition to improved glymphatic function in humans remains limited.

Targeting immune responses and neuroinflammation has also been proposed as a therapeutic strategy. β-hydroxybutyrate has also been shown to inhibit microglial activation, reduce neuroinflammation, and enhance glymphatic drainage in a mouse model of subarachnoid hemorrhage.^115^

Several additional therapeutic strategies have been proposed. Neuromodulation is another promising approach; continuous theta burst stimulation (cTBS), a form of repetitive transcranial magnetic stimulation, improved glymphatic function by improving AQP4 polarization in animal studies^116^ and in a small cohort of cSVD patients,^117^ potentially via modulation of glial activation.^118^ Another neuromodulatory approach to enhance glymphatic function is focused ultrasound combined with microbubbles, as microbubbles oscillation amplifies vessel wall motion and fluid convection and is shown to improve the clearance of beta-amyloid in animal models.^119,120^

## Summary

cSVD and glymphatic dysfunction share common vascular risk factors. Accumulating evidence suggests cSVD impairs glymphatic function through structural and functional alterations of small vessels. Conversely, glymphatic dysfunction may also contribute to the development and progression of cSVD, indicating a potentially bidirectional relationship.

If impaired glymphatic dysfunction does play a causal role of cSVD, this raises the exciting possibility that improving CSF dynamics could be a novel treatment for cSVD. Potential approaches include cardiovascular risk modification, improving sleep, as well as a number of novel therapeutic approaches. Due to the inter-relationships between cSVD, neurodegenerative disease such as AD, and CSF dynamics, such treatments may also be useful in neurodegenerative disease, and particularly in mixed dementia, the most common dementia type in the community.

## Road map for future research

- Further validation of non-invasive glymphatic imaging markers against reference standards, such as intrathecal gadolinium-enhanced MRI, in larger and well-characterized cSVD cohorts.
- Development of more robust non-invasive techniques with reduced susceptibility to confounding factors, including methods targeting meningeal lymphatic function and emerging approaches such as CSF-STREAM.
- Longitudinal studies to clarify the temporal sequence and relative contributions of distinct glymphatic components—including CSF inflow, interstitial fluid exchange, and waste clearance—to vascular injury, cSVD progression, vascular brain damage, and subsequent vascular cognitive impairment and dementia.
